# Key advances in vaccine development for tuberculosis—success and challenges

**DOI:** 10.1038/s41541-023-00750-7

**Published:** 2023-10-13

**Authors:** Rocky Lai, Abiola F. Ogunsola, Tasfia Rakib, Samuel M. Behar

**Affiliations:** Department of Microbiology and Physiological Systems, University of Massachusetts Chan Medical School, Worcester, MA USA

**Keywords:** Vaccines, Vaccines

## Abstract

Breakthrough findings in the clinical and preclinical development of tuberculosis (TB) vaccines have galvanized the field and suggest, for the first time since the development of bacille Calmette-Guérin (BCG), that a novel and protective TB vaccine is on the horizon. Here we highlight the TB vaccines that are in the development pipeline and review the basis for optimism in both the clinical and preclinical space. We describe immune signatures that could act as immunological correlates of protection (CoP) to facilitate the development and comparison of vaccines. Finally, we discuss new animal models that are expected to more faithfully model the pathology and complex immune responses observed in human populations.

## Introduction

Tuberculosis (TB) stands as a prominent cause of death from a single infectious agent, currently being surpassed in 2020–2022 only by Sars-CoV2^1^. This disease, caused by the acid-fast bacillus *Mycobacterium tuberculosis*, was identified by Robert Koch in 1882, and has seen an increase in the death rate to 1.6 million people per year, up from the previously reported rate of 1.4 million deaths per year in 2019, and reversing an overall decrease observed between 2015–2019^1^. Effective TB treatments are expensive and lengthy, often requiring 6–9 months of antibiotic therapy, depending on the regiment used. Furthermore, the emergence of multidrug resistant and extensively drug resistant forms of *M. tuberculosis* have increased the urgency of developing an effective vaccine as an arm of the global TB eradication program.

The sole licensed TB vaccine to date is bacille Calmette-Guérin (BCG), which was first developed in 1921 and is still widely used throughout the world^2^. As the only vaccine approved by the World Health Organization, BCG is generally safe across all age groups and communities, with the exception HIV-infected and other immunocompromised individuals. BCG provides significant protection against disseminated and meningeal TB when administered soon after birth, and protection lasts for up to 10 years^3–5^. However, BCG’s efficacy against pulmonary TB in adults and adolescents varies greatly and has proven variable in its ability to reduce the incidence of pulmonary TB.

Why is BCG efficacy against adult pulmonary TB variable? The success of BCG at preventing childhood TB would suggest that its efficacy wanes with age, although this can vary greatly between studies^6–8^. BCG is also less effective in endemic areas. Exposure to environmental non-tuberculous mycobacteria (NTM) has been proposed as a cause for the variable efficacy of BCG, especially in tropical regions in which BCG efficacy is inversely proportional to the prevalence of NTM^9–12^. A recent study showed that mucosal exposure to NTM elicits protective B cell-mediated immunity and enhances protection against *M. tuberculosis* challenge of mice^13^. If environmental exposure to NTM provides protection against *M. tuberculosis* in some populations, any benefit of BCG vaccination might be nullified in this setting. Differences in preparation (e.g., cultivation) and genetic variability (i.e., mutations) in BCG strains used around the world has been suggested to contribute to its variable efficacy ^14^; however, meta-analyses do not support this hypothesis^15,16^. Finally, variable BCG efficacy could result from a plethora of host factors that vary among different geographical locations including cultural practices, host genetics and environment exposures. However, testing the contribution of these different factors in human populations, and until recently, even in animal models (see below), has been extremely difficult.

The ineffectiveness of BCG against pulmonary TB has prompted many groups to design alternative vaccines to enhance or replace BCG. Numerous vaccine candidates developed over the years are in various stages of clinical development (Table 1), and many more are in preclinical development. Nonetheless, the goal of achieving a universally effective TB vaccine faces significant challenges. In particular, insufficient global investment in TB vaccine research and development hampers progress in this field^17^. Consequently, the selection of candidates for clinical efficacy trials requires careful and systematic consideration, given limited global financial resources. Despite these obstacles, we have recently witnessed exciting advancements in TB vaccine development. Two different TB vaccine trials had demonstrated success in reducing *M. tuberculosis* disease in high-risk populations^18^ or in preventing sustained *M. tuberculosis* infection^19^ (see below). Moreover, for the first time in the history of TB vaccine development, two distinct preclinical vaccine candidates achieved sterilizing immunity in a non-human primate (NHP) model of TB^20,21^. These studies show that a highly effective TB vaccine is feasible. However, the crucial question remains: What will propel us to the finish line? The identification of a reliable correlate of protection (CoP) capable of clearly identifying immune responses that associate with vaccine efficacy would greatly advance this goal. While assessing correlates of protection in human trials is challenging due to high costs, logistical requirements, and analytical challenges, such studies are underway. In addition, preclinical NHP and mouse models are generating immune correlates, which can be potentially translated to human populations. This review provides an overview of the different types of TB vaccines; discusses immune correlates of protection that could facilitate identification of effective vaccines, and highlights new insights gained from preclinical animal models that can be translated to human populations.

**Table 1:** Categories of TB vaccines that are undergoing clinical or preclinical development.

Type	Advantages	Disadvantages	Examples
Whole cell live vaccines	• Generally safe • Generally given as a single dose • Broad range of antigens • Trigger diverse immune responses • Mimic immunity elicited by *M. tuberculosis*	• Not advised for immunocompromised persons	BCG revaccination Intravenous BCG MTBVAC (*M. tuberculosis*) VPM1002 (BCG)
Inactivated whole cell vaccines or lysates	• Safe for immunocompromised persons • Contain diverse repertoire of antigens • Can be used as a prophylactic and post-exposure vaccine	• No immunity to antigens produced during live infection • Generally requires boosting	DAR901 (*M. obuense*) V7 (*M. vaccae*) Immunovac (*M. indicus pranii*) RUTI (*M. tuberculosis*)
Protein subunit and adjuvant	• Safe for immunocompromised persons • Trigger CD4 T cell and antibody responses	• Immune responses are generated to a limited number of antigens • CD8 T cell responses are not elicited • Requires boosting • Requires an adjuvant	AEC/BC02 GamTBvac ID93/GLA-SE M72/AS01E
Viral vectored vaccines	• Generally safe • Trigger robust immune responses • Stimulate CD4, CD8, and antibody responses • Do not require adjuvant	• Strong immune responses to the viral component limit their repeated use • Preexisting immunity against the viral vector limits the use of some vectors	MVA85A AdAg85A RhCMV/TB
mRNA vaccine	• Safe for immunocompromised persons • Speed and flexibility of production • Can elicit different types of immune responses • No pre-existing immunity components	• Like subunit vaccines, immune responses are generated to a limited number of antigens • Relatively untested in the TB field.	ID91

## Current vaccine strategies

There are 21 candidates in the global TB vaccine pipeline that are undergoing in clinical evaluation (Table 1), including vaccines designed for prophylactic and therapeutic use. The details of the different vaccines have recently been reviewed ^22–25^; what follows is a brief overview of the different vaccine categories:

### Live whole-cell vaccines

Whole cell vaccines use an attenuated form of the pathogen to stimulate immune responses which closely mimics the responses elicited by the pathogen. Such vaccines elicit a broad range of immune responses since they contain many more antigens than subunit or viral vector vaccines that typically express few mycobacterial antigens^26^. Live whole cell vaccines rely on viable organisms as a delivery platform. However, live attenuated vaccines can potentially cause disease in individuals with weakened immune systems such as people living with HIV/AIDS, who are at much higher risk of developing active TB disease^26^. The best example of a live whole cell vaccine is BCG, which is an attenuated form of *Mycobacterium bovis*, a mycobacterial species that is closely related to *M. tuberculosis*. BCG has many advantages as a vaccine. As it is genetically related to *M. tuberculosis*, its antigens are conserved. As the cell biology of intracellular infection is similar, both BCG and *M. tuberculosis* elicit both CD4 and CD8 T cell responses and stimulate antibody production. During its attenuation, deletions of genomic regions removed its major virulence factors; consequently, BCG is safe in immunocompetent hosts. With the advent of being able to genetically manipulate mycobacterial species, it became possible to manipulate BCG, with the goal of improving its efficacy in preventing TB disease. The safety record and clinical familiarity of BCG has prompted development of recombinant BCG strains to improve the immune responses elicited by vaccination^26^. The VPM1002 vaccine is a promising example that is in Phase 3 clinical trial. VPM1002 is based on BCG, which has been modified by introducing the gene encoding listeriolysin O (LLO) from *Listeria monocytogenes* and deleting the BCG urease C gene, to alter the pH of phagosomes containing BCG and promote LLO activity^27^. The LLO protein promotes translocation of *L. monocytogenes* from the phagosome into the cytosol of the infected macrophages. When expressed in BCG, LLO is thought to increase the translocation of bacterial antigens into the cytosol, although it also promotes apoptosis, autophagy, and inflammasome activation^28^.

These changes in BCG elicit stronger CD4 and CD8 T cell responses in both preclinical and clinical settings^29,30^. An emerging strategy is attenuation of *M. tuberculosis* itself. The rationale is that there could be key antigens present in *M. tuberculosis* that are absent from BCG that elicit protective immunity. In clinical development, MTBVAC, is an attenuated mutant of *M. tuberculosis* created by the deletion of two independent virulence genes (phoP and fadD26). These deletions significantly reduce the virulence of *M. tuberculosis* while maintaining its ability to induce protective immune response against TB^31^. Finally, recent NHP studies have suggested that the efficacy of BCG itself may be improved when delivered through the intravascular (IV) route^21^. Although the current mechanism by which IV BCG achieves protection in rhesus macaques is still unclear, these findings argue against the speculation that BCG lacks antigens that elicit protective immune responses. Although deploying universal IV BCG vaccination does not seem feasible, it is currently the most efficacious vaccine strategy, and highlights the route of immunization as an important determinant of vaccine-induced protection.

### Inactivated whole-cell vaccines

Inactivated whole cell vaccines work very much the same way as the live whole cell vaccines in the sense that the entire organism stimulates a broad range of immune responses. However, as the organism has been killed, it cannot cause disease and is considered much safer than live vaccines, particularly for immunocompromised people. With that said, they still contain a wide variety of bacterial components which trigger inflammation, such as injection site reactions and other potential side effects. A highly studied example is the vaccine DAR-901, which is inactivated *M. obuense*. Using a heterologous vaccination approach (i.e., a booster that differs from the initial vaccine), adolescents that were vaccinated with BCG as infants are administered DAR-91 to boost BCG memory immune responses. However, in a phase 2b clinical trial the DAR-91 vaccine was unable to prevent *M. tuberculosis* infection in BCG-vaccinated adolescents in Tanzania^32^. Other inactivated whole cell vaccines such as RUTI and V7, are being tested as therapeutic vaccines, designed to be given as an adjunct to standard antibiotic treatment. RUTI is derived from liposomal fragments of *M. tuberculosis* and has completed a phase 2 clinical trial^33^. Similarly, the oral V7 vaccine is derived from inactivated *M. vaccae* and has recently completed a phase III clinical trial, where it was found to be safe and helped to reduce hepatotoxicity of TB drugs^34^.

### Subunit vaccines

Instead of targeting the entire pathogen, protein subunit vaccines are designed to elicit immune responses to specific antigens from the pathogen. Most subunit TB vaccines target certain proteins from *M. tuberculosis* and as such the breadth of immune response is generally much narrower, which makes the choice of antigenic target particularly important. In general, one selects antigens that are known to elicit protective immunity in most people. Because they are not viable, subunit vaccines have a greater safety profile and can be given to immunocompromised individuals without risk of infection. Other advantages include greater control over the dose and vaccination regimen. As subunit vaccines lack “pathogen-associated microbial patterns,” which trigger activation of innate immune responses, they require adjuvants to elicit strong immune responses against the target antigen, making the choice of adjuvant an important consideration in subunit vaccine design. Examples of these vaccines include H4:IC31^19^ and M72/AS01^35^, which have recently completed phase II clinical trials and are discussed further below. Other candidates, such as the H56:IC31^36^ and ID93/GLA-SE^37^ have completed early phase clinical trials and have proven to be safe and immunogenic in human volunteers.

### Viral vector vaccines

Viral-vectored vaccines occupy an interesting middle ground between whole cell vaccines and subunit vaccines. Conceptually, they resemble subunit vaccines in the sense that they are designed to elicit immune responses against a portion of the pathogen rather than the entire organism. The viruses (i.e., the vector) are engineered to encode genes for *M. tuberculosis* proteins. After immunization with the viral particles, virally infected cells are turned into factories to produce the *M. tuberculosis* proteins and induce a robust immune response without the need of an exogenous adjuvant. This approach leverages decades of experience using attenuated viruses as vaccines (e.g., poliovirus, vaccinia, measles) and takes advantage of the immune system’s strong innate and adaptive immune responses to viruses. Vectors are often chosen from viruses that are naturally tropic to humans, such as adenoviruses (Ad), cytomegaloviruses (CMV) and poxviruses such as the modified vaccinia virus Ankara (MVA)^38^. The choice of vector is a major consideration as it will determine how the antigens are delivered and the adjuvant effect. With that said, many of these viral vectors have extensive safety history and have been demonstrated to be capable of inducing a robust immune response^38^. Examples of virally vectored TB vaccines include AdHu85A^39^ and AdHu35^40^, both of which have completed Phase I clinical trial, and the MVA85A, which has completed a large scale phase II trial in South Africa^41^. In addition, a recently developed CMV-based vector induced sterilizing immunity in a preclinical NHP model (^20^, discussed below). One major downside to the use of viral vectors is the existence of pre-existing immunity to the virus. If a person has preexisting immunity to the vector at the time of vaccination, immune responses to the vector will be boosted, leading to premature clearing of the virus and dampening of the *M. tuberculosis*-specific response. To circumvent this potential problem, vaccine developers are using rare human serotypes (e.g., AdHu35) or viruses from other species (e.g., ChAd68Ag85A and ChAdOx1.85A)^40,42–45^. Many of these viruses are also slated for further clinical development as vaccine vectors.

### mRNA vaccines

mRNA vaccines have been in development since the early 90s and were successfully deployed during the SARS-CoV-2 pandemic^46^. As was the case for the mRNA vaccines developed for SARS-CoV-2, their appeal includes the speed and flexibility of design and production once the relevant genetic information is available^47^. The low complexity of viral genomes makes this relatively straightforward for SARS-CoV-2 and other viruses. In contrast, *M. tuberculosis* has approximately 4000 genes^48^, and studies of the *M. tuberculosis* genome reveal an unappreciated genetic diversity among *M. tuberculosis* strains^49^. Selecting antigen targets for mRNA vaccines, like conventional subunit vaccines, is further complicated by the lack of data concerning which antigens are sampled by MHC and presented by infected cells. However, a recent study by Larsen et al. demonstrated the feasibility of this strategy by adapting their ID91 fusion protein vaccine to the mRNA vaccine platform^50^. Although the protection provided by the mRNA version of ID91 was modest, it elicited T cell responses to a broader repertoire of epitopes than their ID91 protein subunit vaccine. The generation of broad responses is especially important in large human populations with diverse MHC haplotypes and such a feature could be an advantage of mRNA vaccines. Used as part of a heterologous vaccine strategy, mRNA vaccines might be able to increase the diversity of elicited immune responses.

## Recent progress in evaluating TB vaccines in clinical trials

A major roadblock to testing vaccines is defining tractable endpoints. Our inability to detect *M. tuberculosis* in asymptomatic individuals is a major impediment to evaluating the ability of vaccines to prevent infection. While microbiological techniques can detect *M. tuberculosis* in individuals with pulmonary disease, these tests can be confusing and often fail to detect individuals with paucibacillary disease^51^. Tuberculin Skin Tests (TST) offer a quick and cheap alternative but cannot differentiate between *M. tuberculosis* infection and BCG vaccination^52^. The current gold standard in TB diagnostics is the Interferon Gamma Release Assay (IGRA). A subject’s peripheral blood mononuclear cells are stimulated with specific *M. tuberculosis* antigens, and the amount of IFNγ released is measured^53^. Since the antigens used in the assay are expressed by *M. tuberculosis* and not by BCG, it can specifically detect *M. tuberculosis* infection even in BCG vaccinated individuals. However, as it measures T cell responses and not bacilli, it indicates exposure to *M. tuberculosis* and cannot differentiate between individuals who have cleared the infection or are persistently infected.

Although many vaccine candidates have entered the clinical trial space, relatively few candidates have advanced to large scale trials beyond Phase I, i.e., studies designed to evaluate vaccine safety and immunogenicity in a relatively small population. The MVA85A vaccine was one of the first vaccines to enter a large phase IIb efficacy trial in 2013. The vaccine failed to statistically augment protection against TB disease among previously BCG-vaccinated 4–6-month-old infant^41^. Nevertheless, this study demonstrated the feasibility of running large phase efficacy trials in areas with a high TB incidence.

Since the MVA85A trial, significant advancements include the results from two recent studies that have re-galvanized the field. First, the M72/AS01E vaccine was evaluated in a Phase 2b trial to prevent disease in a cohort of 3575 HIV-negative adults with latent TB infection (i.e., people who have immunological evidence of *M. tuberculosis* infection but currently do not have clinical symptoms of TB). In this prevention of disease (POD) trial design, participants were randomly assigned to receive either two doses of M72/AS01E or placebo, with a one-month interval between doses. The participants were followed for three years, and the primary endpoint was microbiologically confirmed active, pulmonary TB without evidence of HIV infection^18^. The primary analysis, conducted two years after the second vaccination, revealed that there were 49.7% fewer cases of active TB among persons vaccinated with M72/AS01E compared to the placebo. All individuals in the M72/AS01E cohort developed IgG responses to the M72 protein by the second month after vaccination, and these responses were sustained up to month 36. Furthermore, 23.5% of the M72/AS01E recipients developed polyfunctional CD4 T cells responses that produced IFNγ, TNF, or IL-2 after the first vaccination, which increased to 53.7% by month 36. These results are exciting as this is the first indication that a subunit vaccine can protect *M. tuberculosis*-infected individuals from progressing to active symptomatic TB.

The second study compared BCG revaccination vs. H4:IC31 in preventing *M. tuberculosis* infection among high-risk adolescents^19^. This randomized, partially blinded trial had a dual purpose of assessing the protective effect of BCG revaccination as well as evaluating a novel recombinant protein vaccine candidate, H4, which contains mycobacterial antigens Ag85B and TB10.4, and a new adjuvant IC31, which signals through TLR9. The primary endpoint was conversion from a negative to a positive IGRA, serving as an indicator of *M. tuberculosis* infection, compared to a placebo control. As these vaccines were being evaluated in IGRA^–^ patients (i.e., an uninfected population), the goal was to evaluate the ability of either vaccine to prevent *M. tuberculosis* infection (i.e., a prevention of infection (POI) trial design). A total of 990 adolescents in the Western Cape of South Africa were enrolled and assigned to three study arms. While neither vaccine arm demonstrated statistically significant protection against IGRA conversion compared to the placebo, BCG revaccination exhibited 45.4% efficacy in preventing sustained IGRA conversion, which was interpreted as preventing latent TB infection. In contrast, H4:IC31 had only 30.5% efficacy, leading to the termination of this arm of the study. Interestingly, BCG revaccination upregulated Th1 and IL-22 producing CD4 T cell responses, along with a modest increase in IFNγ-producing natural killer (NK) cells. Ongoing efforts are underway to validate the efficacy of BCG revaccination in a larger cohort of 1800 South African adolescents (NCT04152161), with an expected completion date in early 2026. These results demonstrate a potential new use for BCG in protecting high-risk populations from becoming infected with *M. tuberculosis*.

While encouraging, these trials highlight the difficulties in evaluating vaccines that prevent TB in human populations. POI can only be measured indirectly through immunological techniques such as IGRA. Serial IGRAs have shown that some individuals transiently have a positive test but then revert to negative. While this is interpreted as preventing infection, the long-term significance is unknown. Another subset of infected people that have a distinct type of immunity and remains IGRA negative^54^. An independent CoP that can be measured in vaccinees would accelerate preclinical and clinical development of TB vaccines.

### Immune correlates of protection

A CoP is a quantifiable feature induced by vaccination that correlates with vaccine-induced protection from infection or resistance to disease. For many approved vaccines, the titer of neutralizing antibodies is an example of a CoP that serves as a reliable metric^55^. A CoP for TB could help discriminate between vaccine strategies and optimize vaccine dose and route and select adjuvants, all before performing large scale efficacy trials. Currently, TB vaccine testing is entirely empirical. Despite its importance, the quest for a definitive CoP for TB has encountered multiple obstacles.

One factor that has hindered the identification of CoPs is the complexity of the immune response involved. Immunity to TB involves the complex interplay of various components of the immune response including, but not limited to, T cells, B cells and innate cells^56–58^, and it has been difficult to identify individual factors that strongly associate with control of *M. tuberculosis*. Given the central role of IFNγ in immunity to TB, it was hypothesized that T cell production of IFNγ could serve as a CoP. However, while IFNγ is necessary for immunity to TB^59,60^, it is not sufficient on its own to provide protection. Animal studies suggest that IFNγ levels are associated with bacterial burden and not bacterial control^61^. Recent studies suggest that CD4-derived IFNγ play a minimal role in the control of pulmonary TB^62^, and several IFNγ-independent immune signatures have been associated with TB control^54^. This suggests that, while crucial for immunity against TB, IFNγ levels are not a useful vaccine CoP.

A unique challenge in CoP identification for TB is the phenomenon of latent infection. Latently infected individuals harbor *M. tuberculosis* without overt signs of active disease^63^. The immunological mechanisms in containing and controlling *M. tuberculosis* during this quiescent phase are not clear. The immunological mechanisms that forestall progression to active disease might fundamentally differ from those needed to prevent initial infection. We need to consider the possibility that distinct immunological features correlate with a vaccine’s ability to prevent initial infection versus halt the progression from latent to active disease. Despite these potential obstacles, there has been progress in identifying immune signatures that could be used as vaccine CoP.

### Correlates of protection from vaccines that promote sterilizing immunity in NHP models

Promising preclinical data shows that TB vaccines can induce sterilizing immunity. A CMV-vectored vaccine (RhCMV/TB) led to a 68% reduction in *M. tuberculosis* infection and disease compared to unvaccinated controls in rhesus macaques^20^. Notably, 14 out of 34 vaccinated animals had no detectable disease, including 10 animals in which no *M. tuberculosis* was detected in any tissues. Another study compared different routes of BCG and found that intravenous (IV) BCG induced the best protection, and 9 out of 10 macaques had no detectable infection^21^. Antigen-specific T1/17 responses were associated with protection in multiple NHP studies^64,65^ including bronchoscope-delivered BCG^66^.

Ironically, the high level of protection conferred by these vaccine regimens makes it challenging to detect cellular or humoral CoP. A follow-up study using different doses of IV BCG revealed that T cell infiltration into the airway correlated with BCG dose^67^. The polyfunctionality of recruited CD4 and CD8 T cells was also influenced by the dose. Interestingly, immune responses in bronchoalveolar lavage (BAL) showed stronger correlation with IV BCG-induced protection than PBMC responses. Multivariate analysis identified several immune signatures strongly associated with protection in BAL, including specific subsets of CD4 T cells producing IFNγ and TNF, the number of NK cells in the airway, CD4 T cells producing IL-17 and TNF, and PPD-specific IgA titers. Furthermore, protected animals exhibited enriched immune signatures such as plasma sCD40L and IL-8, cytokine-producing CD8 T cells, and CD107-positive cytokine-producing Vγ9 T cells.

Both studies hold significant implications for TB vaccine development as they show that vaccines are capable of eliciting sterilizing immunity against TB. In addition, we need to re-evaluate the role of BCG in preventing TB. Interestingly, both strategies make use of replication-competent vaccines. Although the persistence of these vectors may allow these vaccines to achieve a higher antigenic threshold, there is also the potential safety concerns of using a replicative-competent vector, especially in immunodeficient hosts. The compelling evidence that BCG can induce sterilizing immunity challenges the notion that the inconsistent efficacy of BCG vaccination in preventing pulmonary TB is attributable to BCG itself. Moreover, these results dispel the idea that BCG fails to express antigens that are needed to elicit protective immunity. Now, with two highly effective vaccines, RhCMV/TB and IV BCG, both which induce sterilizing immunity in the NHP model, the identification of CoP that can translate to human population should be a priority.

### Antibody responses as a correlate of protection

Many vaccines are successful because they stimulate antibody responses that neutralize extracellular pathogens. In addition, vaccines induce protective antibody responses against intracellular bacterial pathogens including *Salmonella, Shigella* and *Yersinia*^68^. Antibody-dependent mechanisms for controlling *M. tuberculosis* infection are observed, renewing interest in vaccines that elicit antibodies to *M. tuberculosis*. Notably, people with latent TB have distinct antibody profiles compared to those with active TB^69^. These differences can potentially distinguish these two disease states. Furthermore, these differences correlate with Fc receptor capabilities and the antibodies that are most abundant during latent infection correlate with increased control of replicating intracellular bacteria. Preclinical models have also shown that antibodies can be protective. IV administration of an anti-LAM IgG antibody to BALB/c mice, either prior to or at the time of IV *M. tuberculosis* infection significantly reduces the bacterial burden in the lungs and spleens of infected animals and prolongs their survival^70^. Similarly, pre-incubation of *M. tuberculosis* with antibody specific to a *M. tuberculosis* surface antigen prolongs survival of immunocompromised mice. IFN-γ-deficient C57BL/6 mice infected intratracheally with *M. tuberculosis* pre-coated with 9d8 mAb survive longer than a control group using a non-specific mAb. The reason for the enhanced survival is unknown as there was no significant difference in bacterial burden in the lung or spleen between the groups^70,71^.

BCG vaccination in humans generates specific antibodies that are mostly IgG^72^. In a study of 66 infants, PPD-specific serum IgM antibodies rose steadily post-vaccination, whereas PPD-specific serum IgG antibodies began to increase four months post BCG-vaccination^73^. Both primary and secondary intra-dermal BCG vaccination elicits LAM-specific antibodies that enhance the stimulation of cell-mediated immune responses; these antibody titers increase with subsequent vaccine doses. IgA antibody responses are preferentially generated following oral BCG vaccination^74,66^. Humoral responses following BCG vaccination was once thought to be of little importance, however, review of clinical data reveals an association between the antibody titer and a decreased likelihood/prevalence of infection^75^. Although it is well-established that BCG vaccination elicits humoral responses, the data suggesting that these responses are protective are varied and somewhat contradictory^76,77^. However, in the pre-clinical model of the rhesus macaque, the IgM antibody response following BCG vaccination correlates with reduced bacterial burden after challenge with *M. tuberculosis*^78^. This effect was related to the route of BCG administration with IV administration generating more robust antibody titers compared to the intradermal route.

BCG is not the only vaccine that generates antibody responses against *M. tuberculosis*. A DNA vaccine containing the gene encoding the major secretory protein Ag85b generates robust antibody responses as detected by ELISA and is associated with a reduction in the lung *M. tuberculosis* burden^79^. The extent of protection observed was similar to BCG vaccination, and is observed when this vaccine is given intranasally or intramuscularly, suggesting that these routes of administration generate sufficient antibody titers that correlate with protection against subsequent infection C57BL/6 and BALB/c mice^80,81^. These antibody responses are not unique to vaccines generated from secretory proteins. Similar protective efficacy is observed with the Mtb10.4-HspX subunit vaccine and the yeast-expressed recombinant HBHA co-administered with mucosal adjuvant cholera toxin (CT). Mice vaccinated with the Mtb10.4-HspX subunit vaccine, produced enhanced IgG antibodies compared to BCG vaccinated animals, but also an increased IFN-γ and IL-17 response from stimulated splenocytes. When the Mtb10.4-HspX subunit vaccine was used to boost BCG vaccination, there was a marked reduction in the number of *M. tuberculosis* lung lesions and corresponding decrease in bacterial burden^82^. Intranasal administration of HBHA/CT elicited a significant abundance of mycobacterial specific antibodies compared to the control or lack of adjuvant. Additionally, HBHA/CT was shown to increase T cell proliferation and IFN-γ production. Together, these immune responses correlate with decreased splenic bacterial burden following subsequent BCG challenge^83^.

### Trained innate immunity as a correlate of protection

Although vaccine development has generally focused on strategies to elicit adaptive immune responses, there is now an appreciation that innate immune responses triggered by vaccines can mediate protection, independent of adaptive immune responses. In 1941, some nursing students remained uninfected despite being heavily exposed to *M. tuberculosis*, suggesting that factors other than adaptive immunity prevented infection^84^. Similarly, 13 out of 66 sailors remained TST negative despite sharing quarters with others that had active pulmonary TB^85^. Although the absence of TST responses does not rule out a potential role for non-conventional T cells in protection^86^, these studies raise the possibility that some people can be protected against TB without requiring an adaptive memory T cell response. The implication is that protection was mediated through innate immune mechanisms.

BCG vaccination leads to protection against *M. tuberculosis* but also against non-targeted pathogens such as *Candida albicans* and *Staphylococcus aureus*^87,88^. One mechanism by which BCG can stimulate protection against related and unrelated infections is by educating the innate immune compartment, a process termed “trained immunity”. Trained immunity can also be induced by stimulus other than BCG^89,90^. Epigenetic changes of innate cells are thought to be the major mechanisms by which trained immunity develops^87^, which leads to long-term transcriptional modification and the development of immunological memory in the innate immune compartment^91^. Indeed, some investigators believe that epigenetic changes are a prerequisite of trained immunity.

Trained immunity is now recognized as a potential CoP against *M. tuberculosis* infection. BCG vaccination induces trained innate immunity, which may contribute to early clearance of *M. tuberculosis*^91,92^. In a randomized trial conducted in South Africa to evaluate the impact of BCG revaccination, revaccinated individuals had a reduced risk of sustained IGRA conversion compared to the placebo group^19^. Furthermore, revaccinated subjects experience fewer upper respiratory tract infections, a characteristic of BCG-induced trained immunity. Another study of household contacts in Indonesia reveals significant differences in immune signatures between non-converters (those who remained IGRA negative for over 14 weeks) and IGRA converters^93^. There is a concomitant decrease in frequencies of innate cells (monocytes, granulocytes, and innate-like T cells) in non-converters while no such decrease occurs in the IGRA-converters, suggesting that the non-converters eliminate infection while there is ongoing inflammation caused by *M. tuberculosis* infection in IGRA-converters. Stimulation of whole blood with *Escherichia coli* showed higher levels of cytokines, including TNF, IL-6, and IL8, in non-converters vs. IGRA converters, another feature of trained innate immunity. Similarly, a study in the Netherlands used PBMCs from recently *M. tuberculosis*-exposed or unexposed subjects to show that the former have an enhanced capacity to control *M. tuberculosis* infection in vitro, accompanied by increased levels of TNF, IL-1β, and IL-6, which act as a signature of BCG-induced trained immunity^94^. This response is dependent on CXCR3 signaling and the frequency and activity of non-classical CD14^dim^ monocytes expressing CXCL10, which is the contracting cell population observed in early clearers in the Indonesian study^93^.

Studies with β-glucan, which also induces trained innate immunity, support the concept of training as a CoP against *M. tuberculosis*. In vitro treatment of human monocytes with β-glucan stimulates TNF, IL-1β, and IL-6 production, and these monocytes have greater resistance to *M. tuberculosis* infection^89^. In mice, intraperitoneal administration of β-glucan prior to *M. tuberculosis* infection significantly reduces lung bacterial burden and improves survival. These effects are attributed to the shift of hematopoietic stem and progenitor cells towards myelopoiesis and the initiation of IL-1β signaling, which facilitates the transcription of anti-*M. tuberculosis* genes. Notably, IL-1β itself induces trained immunity^95^, as macrophages derived from monocytes that were treated with IL-1β have enhanced anti-*M. tuberculosis* capacity^96–98^.

Additional evidence supporting the role of trained immunity in protection against *M. tuberculosis* infection comes from studies using mouse parabiosis and adoptive transfer experiments^99^. IV BCG administration results in lower bacterial burden in target organs, and bone marrow-derived macrophages isolated from these mice exhibit lower bacterial burden and increased proinflammatory signatures compared to control groups in response to *M. tuberculosis* infection. Similarly, β-glucan-induced trained immunity confers protection against *M. tuberculosis* infection, with intraperitoneal β-glucan treatment leading to lower lung bacterial burden and improved survival in mice. IV BCG also led to changes in the transcriptional landscape of the hematopoietic stem cell (HSC), resulting in enhanced myelopoiesis at the expense of lymphopoiesis^99^. This contrasts with the effects of *M. tuberculosis* itself where it actively suppresses myelopoiesis and induces cell death in myeloid progenitors through a type 1 IFN cascade^100^.

Despite these promising findings, several unanswered questions remain in the field of trained immunity. A major challenge is the substantial interindividual variability observed among responders and non-responders. Host genetics is likely to play a significant role, as polymorphisms in genes encoding proteins involved in the IL-1β, glycolysis, and autophagy pathways are associated with differential responses. An important consideration is how to distinguish trained immunity stimulated by infection, from changes elicited by vaccination, sterile triggers, or the host microbiota.

## Using novel models to overcome challenges in preclinical TB vaccine development

Various animal models are used to investigate TB and TB vaccine responses including mice, guinea pigs, rabbits, cattle, and NHP^101^. None of these models faithfully reproduce all the features of human TB, although all models have generated important insights into the pathogenesis and treatment of disease. Importantly, no animal species can eliminate *M. tuberculosis* after primary infection. Among these different species, the NHP model most closely resembles human TB, and important discoveries have been made using rhesus and cynomolgus macaques. However, the model is extremely resource-intensive and ethical and financial constraints limit its use. In contrast, mice are widely used because of their ease of handling, cost-effectiveness, and the availability of reagents. Mouse models offer the advantage of genetic tractability and suitability for mechanistic studies. Their relatively short gestation period, ability for multiple births, and shorter lifespan enable the completion of longitudinal studies in a relatively short time frame. While there are immune features that are unique to mice or humans, the components, organization, and working of the murine and human immune systems are sufficiently alike to make the mouse a useful model.

A criticism of the mouse TB model is that it does not faithfully reproduce the pathological features observed in humans, particularly the formation of granuloma structures. Notably, two frequently employed inbred mouse strains, C57BL/6 and BALB/c, do not develop necrotizing lesions that are frequently observed in people with active pulmonary disease^102,103^. These are also among the most resistant inbred mouse strains to *M. tuberculosis* infection, which can make it difficult to identify conditions that can improve their inherent resistance^104^. C57BL/6 mice are frequently chosen to use in mouse studies because of the numerous genetic tools readily available in the C57BL/6 genetic background. However, C57BL/6 mice have limited MHC diversity as it only expresses a single class II MHC molecule and is missing a class I MHC gene^104^. In contrast, other inbred strains such as C3HeB/FeJ mice develop necrotizing lesions resembling those seen in humans and are beginning to be used more frequently^58,105–107^. Given the advantages of the mouse model to test vaccines, identify CoP, and perform mechanistic studies, there is great interest on how to improve its usefulness.

### Using genetically diverse mouse models

Fully leveraging the available genetic diversity available in the mouse species can improve its relevance and replicate key human-like pathological features. To address the lack of genetic diversity in the mouse model, the mouse genetics community developed a resource that captures the genetic diversity of *Mus musculus*. Starting with eight founder strains that represent the three *Mus musculus* subspecies and include wild-derived strains, an eight-way funnel breeding scheme was used to create progeny with random assortment of the founder genomes^108^. This led to the creation of two distinct mouse resources, Collaborative Cross (CC) mice and Diversity Outbred (DO) mice.

Each of the ~70 CC mouse strains has a unique genome that is a random recombination of the eight founders and collectively captures the genetic diversity observed in outbred population. As each individual CC strain is also inbred, the CC mice retains the reproducibility of classic inbred strains and allows for unlimited experiments with the same genotype to allow comprehensive mechanistic analyses. Previous studies have shown variation in susceptibility to TB among CC strains and differences in protection provided by the BCG vaccine.^109–111^. These data show that that host genetics influence vaccine-mediated protection, making CC strains a good model for investigation. To understand how host variation affects BCG-induced immunity against TB, 24 CC strains were vaccinated and challenged with TB^112^. BCG conferred significant protection in only 13 of the 24 strains. Vaccination resulted in changes in T cell responses in protected strains but not in unprotected ones. Some protected CC strains developed Th1/17 signatures, which have been associated with protective immunity in other studies and species^21,64,66^, indicating that they may represent a useful marker of protective vaccine responses.

The DO mice originate from the same eight founder strains as the CC mice. In contrast to CC mice, they are maintained as a truly outbred population. BCG vaccination of DO mice leads to enhanced survival of the population after challenge with *M. tuberculosis*^113^. The heterogeneity inherent in DO mice facilitates high-resolution gene and quantitative trait locus mapping, along with the detection of divergent or rare phenotypes. In addition, a wide range of pathological features have been described in the DO mice, which allows for the study of pathological features in a genetically diverse background^106,114^.

### Low and Ultra-low-dose mouse models

One hypothesis to explain why vaccines underperform in the mouse TB model is that the bacterial inoculum is too high. While the inoculum typically used in mice has steadily fallen during the past three decades, from 10^6^ (administered IV) to 25–50 (delivered by aerosol) CFU, this small dose could still overwhelm the murine immune system. The typical aerosol inoculum causes progressive inflammatory disease and poorly organized granulomatous structures^58^. Although the infectious dose in people is unknown, some have speculated that is it low^115–117^. As human TB infection encompasses a wide range of outcomes, from potential eradication to long-term asymptomatic containment to active disease with a high burden^63^, one possible explanation for the differences in outcome could be variation in the infectious inoculum. To address this possibility, Plumlee et al used an ultra-low dose (ULD) *M. tuberculosis* infection mouse model, aiming for an inoculum of 1–3 bacilli^118^. This approach results in increased diversity in infection outcomes and the formation of well-defined granulomatous structures, which are typically absent in classic C57BL/6 mice infected with a conventional inoculum. With this ULD model, a blood RNA signature was identified that could predict the severity of TB disease in NHP. Recently, the ULD mouse model was used to evaluate the protective efficacy of BCG vaccination, with the important finding that BCG could prevent infection in some of mice^119^. Therefore, the ULD model could represent an important advance in the use of mice to model TB vaccine responses.

## Lessons learned: how to design the perfect vaccine

What does this all mean for TB vaccine design? *M. tuberculosis* evolved with humans for the past 15,000 years, and presumably, it evolved to avoid, evade, and subvert human immune responses, and has emerged as a successful human pathogen^120^. Nevertheless, as only 5–10% of infected people develop disease, the human immune system can eliminate or contain *M. tuberculosis* infection in most cases. The question of why certain people develop disease has not been definitively answered. Mendelian Susceptibility to Mycobacterial Disease (MSMD) is a syndrome caused by mutations in different genes, mostly in the generation of Th1 responses and are an inherited cause of susceptibility to disease^121^. Although MSMD cases are rare, they provide a basis to believe that susceptibility in the general population could be polygenic, an idea that is supported by varying susceptibility among inbred mouse strains and documented in a limited number of human studies^110,122^. A genetic basis for susceptibility to infection provides theoretical barriers to vaccination as it is uncertain whether vaccination could generate protective immunity in people with a genetic susceptibility to TB. For example, many individuals with MSMD were identified because they developed disseminated BCG following BCG vaccination. We know that human susceptibility to *M. tuberculosis* can also be acquired, mostly by comorbid conditions that impair cell mediated immunity. HIV infection leading to AIDS is an extreme and well-documented example^123^. Other causes include malnutrition, alcoholism, diabetes, and immunosuppressive medications. Protective immunity would be expected to wane in people with impaired cell mediated immunity.

Bacterial factors also need to be considered. Inoculum size and repeated exposure may overwhelm the capacity of the immune system to protect the host. Bacterial virulence factors, including drug resistance, may reflect the ongoing evolution of *M. tuberculosis*, driven by selective pressure to escape immune control. Given these challenges, it will be important to design a vaccine that leverages all the components of the immune system. An important decision is whether to use whole cell vaccines or ‘functional’ subunit vaccines (i.e., protein or viral vectored vaccines). The former elicits diverse immune responses (e.g., many antigens, antibody, and T cell responses, various types of T cells) but there is no control over antigen expression (e.g., timing, amount). Subunit vaccines provide excellent control over antigen dose and timing, but the breadth of the immune response is limited (e.g., primarily CD4 T cells and antibodies to a limited number of antigens). Still, both approaches have been successful in achieving sterilizing immunity in the NHP model.

Given the success of IV BCG and RhCMV/TB in achieving sterilizing immunity, mining the associated datasets for possible immune CoP that can be validated in either clinical trial or small animal models could be a fruitful endeavor. Indeed, Th1/17 cells have been identified in IV BCG NHP models as well as mouse models of BCG vaccination^112,124^, and have been identified in patients with latent TB. However, it is unclear how well these responses correlated with protection in human populations and whether the induction of these responses (i.e., by vaccination) could have unintended side effects. For example, Th1/17 responses are associated with autoimmunity^125^.

The recognition that the innate and antibody compartments, as well as non-classical T cell responses, contribute to protective immunity against *M. tuberculosis* indicates that an ideal vaccine would activate multiple immune compartments in addition to conventional CD4 T cells. New data shows that T cell responses modulate the innate immune compartment as a mechanism of vaccine-mediated protection. Specifically, IFNγ production by T cells following vaccination reprograms the myeloid compartment to deliver enhanced resistance against subsequent infection^90,126,127^. While CD4 T cells are generally acknowledged to be crucial for protection, CD4 T cells have a variety of functions. While they can act as direct effectors to activate *M. tuberculosis*-infected macrophages, they also provide help to CD8 T cells and to B cell and antibody responses. A “systems immunology” approach is needed to evaluate the interplay between various immune compartments stimulated by vaccination.

A further consideration is the use of a multi-species approach for the identification of immune correlates. Recent achievements in NHP and mouse models have indicated that a multi-host species strategy may be the most effective path forward. This approach entails leveraging data obtained from animal models to validate targets and strategies in human studies. Recognizing the potential of this strategy, several collaborative efforts funded by the National Institutes of Health are now integrating data from small animal species, NHP, and humans for this purpose^128^. Computational approaches that have already been applied to other disease models^129,130^ can be applied to TB datasets from mouse, NHP, and human studies to identify immune pathways that are common between the datasets, which can then be validated in follow-up studies. Ultimately, the identification of immune CoP should lead to the discovery of crucial mechanisms of protection, which will improve the design, implementation, and evaluation of TB vaccines.

Finally, there is still a pressing need to identify new antigens that can be vaccine targets. T cells remain a crucial aspect of host immunity against TB, and the choice of antigen will likely play a key role in the effectiveness of vaccines, especially for vaccine strategies that target only a subset of the *M. tuberculosis* proteosome. Recent evidence from our lab has suggested that TB10.4, a commonly selected target for TB subunit vaccines, may in fact be a decoy antigen by inducing antigen specific CD8 T cells that poorly recognize infected macrophages^131–133^. A similar phenomenon has been described for an immunodominant epitope for CD4 T cells within the ESAT-6 protein^134^. Identifying antigens that are presented by infected cells early during infection would seem to be ideal antigens for vaccines.

## Conclusion

The results from the M72/AS01 and BCG revaccination trials show that the development of a highly effective TB vaccine is not an insurmountable challenge. TB vaccines can be tested in clinical trials and positive outcomes can be detected. As there is limited capacity for testing new vaccine candidates in late-stage clinical trials, there is still an urgent need for preclinical models that can help select the most promising candidates for further development. Given the practical constraints of conducting large-scale POD trials in humans, both in terms of cost and logistics, a major advance would be the identification of a validated immune CoP that could replace the need to perform outcome studies for each vaccine candidate. By adopting such an approach, TB researchers are developing innovative vaccination strategies approaches and novel ways to assess protection for a disease that has afflicted humanity since ancient times.

## References

[1] World Health Organization. Global Tuberculosis Report 2022. Licence: CC BY-NC-SA 3.0 IGO (Geneva: World Health Organization, 2022).

[2] Ahmed A (2021). A century of BCG: impact on tuberculosis control and beyond. Immunol. Rev..

[3] Abubakar I (2013). Systematic review and meta-analysis of the current evidence on the duration of protection by bacillus Calmette-Guerin vaccination against tuberculosis. Health Technol. Assess..

[4] Hart PD, Sutherland I (1977). BCG and vole bacillus vaccines in the prevention of tuberculosis in adolescence and early adult life. Br. Med J..

[5] Mangtani P (2014). Protection by BCG vaccine against tuberculosis: a systematic review of randomized controlled trials. Clin. Infect. Dis..

[6] Aronson NE (2004). Long-term efficacy of BCG vaccine in American Indians and Alaska Natives: a 60-year follow-up study. JAMA.

[7] Nguipdop-Djomo P, Heldal E, Rodrigues LC, Abubakar I, Mangtani P (2016). Duration of BCG protection against tuberculosis and change in effectiveness with time since vaccination in Norway: a retrospective population-based cohort study. Lancet Infect. Dis..

[8] Whittaker E, Nicol MP, Zar HJ, Tena-Coki NG, Kampmann B (2018). Age-related waning of immune responses to BCG in healthy children supports the need for a booster dose of BCG in TB endemic countries. Sci. Rep..

[9] Palmer CE, Long MW (1966). Effects of infection with atypical mycobacteria on BCG vaccination and tuberculosis. Am. Rev. Respir. Dis..

[10] Poyntz HC (2014). Non-tuberculous mycobacteria have diverse effects on BCG efficacy against Mycobacterium tuberculosis. Tuberculosis..

[11] Verma D, Chan ED, Ordway DJ (2020). Non-tuberculous mycobacteria interference with BCG-current controversies and future directions. Vaccines.

[12] Brandt L (2002). Failure of the Mycobacterium bovis BCG vaccine: some species of environmental mycobacteria block multiplication of BCG and induction of protective immunity to tuberculosis. Infect. Immun..

[13] Dutt TS (2022). Mucosal exposure to non-tuberculous mycobacteria elicits B cell-mediated immunity against pulmonary tuberculosis. Cell Rep..

[14] Behr MA (2002). BCG–different strains, different vaccines?. Lancet Infect. Dis..

[15] Brewer TF, Colditz GA (1995). Relationship between bacille Calmette-Guerin (BCG) strains and the efficacy of BCG vaccine in the prevention of tuberculosis. Clin. Infect. Dis..

[16] Colditz GA (1994). Efficacy of BCG vaccine in the prevention of tuberculosis. meta-analysis of the published literature. JAMA.

[17] TAG. Tuberculosis Research Funding Trends 2005–2021 (2022).

[18] Tait DR (2019). Final analysis of a trial of M72/AS01(E) vaccine to prevent tuberculosis. N. Engl. J. Med..

[19] Nemes E (2018). Prevention of M. tuberculosis infection with H4:IC31 vaccine or BCG revaccination. N. Engl. J. Med..

[20] Hansen SG (2018). Prevention of tuberculosis in rhesus macaques by a cytomegalovirus-based vaccine. Nat. Med..

[21] Darrah PA (2020). Prevention of tuberculosis in macaques after intravenous BCG immunization. Nature.

[22] Scriba TJ, Netea MG, Ginsberg AM (2020). Key recent advances in TB vaccine development and understanding of protective immune responses against Mycobacterium tuberculosis. Semin. Immunol..

[23] Dockrell HM, McShane H (2022). Tuberculosis vaccines in the era of Covid-19—what is taking us so long?. EBioMedicine.

[24] Kaufmann SHE (2021). Vaccine development against tuberculosis over the last 140 years: failure as part of success. Front. Microbiol..

[25] Andersen P, Scriba TJ (2019). Moving tuberculosis vaccines from theory to practice. Nat. Rev. Immunol..

[26] Scriba TJ (2016). Vaccination against tuberculosis with whole-cell mycobacterial vaccines. J. Infect. Dis..

[27] Grode L (2005). Increased vaccine efficacy against tuberculosis of recombinant Mycobacterium bovis bacille Calmette-Guerin mutants that secrete listeriolysin. J. Clin. Invest..

[28] Nieuwenhuizen NE (2017). The recombinant bacille Calmette-Guerin vaccine VPM1002: ready for clinical efficacy testing. Front. Immunol..

[29] Farinacci M, Weber S, Kaufmann SH (2012). The recombinant tuberculosis vaccine rBCG DeltaureC::hly(+) induces apoptotic vesicles for improved priming of CD4(+) and CD8(+) T cells. Vaccine.

[30] Cotton MF (2022). Safety and immunogenicity of VPM1002 versus BCG in South African newborn babies: a randomised, phase 2 non-inferiority double-blind controlled trial. Lancet Infect. Dis..

[31] Arbues A (2013). Construction, characterization and preclinical evaluation of MTBVAC, the first live-attenuated M. tuberculosis-based vaccine to enter clinical trials. Vaccine.

[32] Munseri P (2020). DAR-901 vaccine for the prevention of infection with Mycobacterium tuberculosis among BCG-immunized adolescents in Tanzania: A randomized controlled, double-blind phase 2b trial. Vaccine.

[33] Nell AS (2014). Safety, tolerability, and immunogenicity of the novel antituberculous vaccine RUTI: randomized, placebo-controlled phase II clinical trial in patients with latent tuberculosis infection. PLoS One.

[34] Bourinbaiar AS (2020). Phase III, placebo-controlled, randomized, double-blind trial of tableted, therapeutic TB vaccine (V7) containing heat-killed M. vaccae administered daily for one month. J. Clin. Tuberc. Other Mycobact. Dis..

[35] Van Der Meeren O (2018). Phase 2b controlled trial of M72/AS01E vaccine to prevent tuberculosis. N. Engl. J. Med..

[36] Jenum S (2021). A Phase I/II randomized trial of H56:IC31 vaccination and adjunctive cyclooxygenase-2-inhibitor treatment in tuberculosis patients. Nat. Commun..

[37] Sagawa ZK (2023). Safety and immunogenicity of a thermostable ID93 + GLA-SE tuberculosis vaccine candidate in healthy adults. Nat. Commun..

[38] Hu Z, Lu SH, Lowrie DB, Fan XY (2022). Research advances for virus-vectored tuberculosis vaccines and latest findings on tuberculosis vaccine development. Front Immunol..

[39] Smaill F (2013). A human type 5 adenovirus-based tuberculosis vaccine induces robust T cell responses in humans despite preexisting anti-adenovirus immunity. Sci. Transl. Med..

[40] Churchyard GJ (2015). The safety and immunogenicity of an adenovirus type 35-vectored TB vaccine in HIV-infected, BCG-vaccinated adults with CD4(+) T cell counts >350 cells/mm(3). Vaccine.

[41] Tameris MD (2013). Safety and efficacy of MVA85A, a new tuberculosis vaccine, in infants previously vaccinated with BCG: a randomized, placebo-controlled phase 2b trial. Lancet.

[42] Jeyanathan M (2015). Novel chimpanzee adenovirus-vectored respiratory mucosal tuberculosis vaccine: overcoming local anti-human adenovirus immunity for potent TB protection. Mucosal Immunol..

[43] Afkhami S (2019). Single-dose mucosal immunotherapy with chimpanzee adenovirus-based vaccine accelerates tuberculosis disease control and limits its rebound after antibiotic cessation. J. Infect. Dis..

[44] Afkhami S (2023). Intranasal multivalent adenoviral-vectored vaccine protects against replicating and dormant M.tb in conventional and humanized mice. NPJ Vaccines.

[45] Stylianou E (2015). Improvement of BCG protective efficacy with a novel chimpanzee adenovirus and a modified vaccinia Ankara virus both expressing Ag85A. Vaccine.

[46] Verbeke R, Lentacker I, De Smedt SC, Dewitte H (2019). Three decades of messenger RNA vaccine development. Nano Today.

[47] Chaudhary N, Weissman D, Whitehead KA (2021). mRNA vaccines for infectious diseases: principles, delivery and clinical translation. Nat. Rev. Drug Discov..

[48] Cole ST (1998). Deciphering the biology of Mycobacterium tuberculosis from the complete genome sequence. Nature.

[49] Gagneux S, Small PM (2007). Global phylogeography of Mycobacterium tuberculosis and implications for tuberculosis product development. Lancet Infect. Dis..

[50] Larsen S. E. et al. An RNA-based vaccine platform for use against Mycobacterium tuberculosis. Vaccines. 11. Epub 2023/01/22. 10.3390/vaccines11010130. (2023). 10.3390/vaccines11010130 PMC9862644 36679975

[51] Steingart KR (2006). Fluorescence versus conventional sputum smear microscopy for tuberculosis: a systematic review. Lancet Infect. Dis..

[52] Farhat M, Greenaway C, Pai M, Menzies D (2006). False-positive tuberculin skin tests: what is the absolute effect of BCG and non-tuberculous mycobacteria?. Int J. Tuberc. Lung Dis..

[53] Mazurek GH, Centers for Disease Control and Prevention (2010). Updated guidelines for using Interferon Gamma Release Assays to detect Mycobacterium tuberculosis infection - United States, 2010. MMWR Recomm. Rep..

[54] Lu LL (2019). IFN-γ-independent immune markers of Mycobacterium tuberculosis exposure. Nat. Med..

[55] Khoury DS (2021). Neutralizing antibody levels are highly predictive of immune protection from symptomatic SARS-CoV-2 infection. Nat. Med..

[56] Nunes-Alves C (2014). In search of a new paradigm for protective immunity to TB. Nat. Rev. Microbiol..

[57] Cooper AM (2009). Cell-mediated immune responses in tuberculosis. Annu. Rev. Immunol..

[58] Cohen SB, Gern BH, Urdahl KB (2022). The tuberculous granuloma and preexisting immunity. Annu Rev. Immunol..

[59] Flynn JL (1993). An essential role for interferon gamma in resistance to Mycobacterium tuberculosis infection. J. Exp. Med.

[60] Cooper AM (1993). Disseminated tuberculosis in interferon gamma gene-disrupted mice. J. Exp. Med..

[61] Nandi B, Behar SM (2011). Regulation of neutrophils by interferon-gamma limits lung inflammation during tuberculosis infection. J. Exp. Med.

[62] Sakai S (2016). CD4 T cell-derived IFN-gamma plays a minimal role in control of pulmonary Mycobacterium tuberculosis infection and must be actively repressed by PD-1 to prevent Lethal disease. PLoS Pathog..

[63] Barry CE (2009). The spectrum of latent tuberculosis: rethinking the biology and intervention strategies. Nat. Rev. Microbiol..

[64] Gideon HP (2022). Multimodal profiling of lung granulomas in macaques reveals cellular correlates of tuberculosis control. Immunity.

[65] Gideon HP (2015). Variability in tuberculosis granuloma T cell responses exists, but a balance of pro- and anti-inflammatory cytokines is associated with sterilization. PLOS Pathog..

[66] Dijkman K (2019). Prevention of tuberculosis infection and disease by local BCG in repeatedly exposed rhesus macaques. Nat. Med..

[67] Darrah PA (2023). Airway T cells are a correlate of i.v. Bacille Calmette-Guerin-mediated protection against tuberculosis in rhesus macaques. Cell Host Microbe.

[68] Casadevall A (2018). Antibody-based vaccine strategies against intracellular pathogens. Curr. Opin. Immunol..

[69] Lu LL (2016). A functional role for antibodies in tuberculosis. Cell.

[70] Hamasur B (2004). A mycobacterial lipoarabinomannan specific monoclonal antibody and its F(ab’) fragment prolong survival of mice infected with Mycobacterium tuberculosis. Clin. Exp. Immunol..

[71] Teitelbaum R (1998). A mAb recognizing a surface antigen of Mycobacterium tuberculosis enhances host survival. Proc. Natl Acad. Sci. USA..

[72] Hoft DF (1999). A double-blind, placebo-controlled study of Mycobacterium-specific human immune responses induced by intradermal bacille Calmette-Guérin vaccination. J. Lab Clin. Med.

[73] Beyazova U, Rota S, Cevheroğlu C, Karsligil T (1995). Humoral immune response in infants after BCG vaccination. Tube. Lung Dis..

[74] Brown RM (2003). Lipoarabinomannan-reactive human secretory immunoglobulin a responses induced by mucosal bacille Calmette-Guérin vaccination. J. Infect. Dis..

[75] Fletcher HA (2016). T-cell activation is an immune correlate of risk in BCG vaccinated infants. Nat. Commun..

[76] Abebe F, Bjune G (2009). The protective role of antibody responses during Mycobacterium tuberculosis infection. Clin. Exp. Immunol..

[77] Glatman-Freedman A, Casadevall A (1998). Serum therapy for tuberculosis revisited: reappraisal of the role of antibody-mediated immunity against Mycobacterium tuberculosis. Clin. Microbiol Rev..

[78] Irvine EB (2021). Robust IgM responses following intravenous vaccination with Bacille Calmette-Guérin associate with prevention of Mycobacterium tuberculosis infection in macaques. Nat. Immunol..

[79] Huygen K (1996). Immunogenicity and protective efficacy of a tuberculosis DNA vaccine. Nat. Med..

[80] Giri PK, Verma I, Khuller GK (2006). Enhanced immunoprotective potential of Mycobacterium tuberculosis Ag85 complex protein based vaccine against airway Mycobacterium tuberculosis challenge following intranasal administration. FEMS Immunol. Med. Microbiol..

[81] Teixeira FM (2006). DNA vaccine using Mycobacterium bovis Ag85B antigen induces partial protection against experimental infection in BALB/c mice. Clin. Vaccin. Immunol..

[82] Kohama H (2008). Mucosal immunization with recombinant heparin-binding haemagglutinin adhesin suppresses extrapulmonary dissemination of Mycobacterium bovis bacillus Calmette-Guérin (BCG) in infected mice. Vaccine.

[83] Niu H (2011). Construction and evaluation of a multistage Mycobacterium tuberculosis subunit vaccine candidate Mtb10.4-HspX. Vaccine.

[84] ISRAEL HL, HETHERINGTON HW, JG ORD (1941). A study of tuberculosis among students of nursing. J. Am. Med. Assoc..

[85] Houk VN, Baker JH, Sorensen K, Kent DC (1968). The epidemiology of tuberculosis infection in a closed environment. Arch. Environ. Health.

[86] Simmons JD (2018). Immunological mechanisms of human resistance to persistent Mycobacterium tuberculosis infection. Nat. Rev. Immunol..

[87] Kleinnijenhuis J (2012). Bacille Calmette-Guerin induces NOD2-dependent nonspecific protection from reinfection via epigenetic reprogramming of monocytes. Proc. Natl Acad. Sci. USA..

[88] Kleinnijenhuis J (2014). BCG-induced trained immunity in NK cells: role for non-specific protection to infection. Clin. Immunol..

[89] Moorlag S (2020). beta-Glucan induces protective trained immunity against Mycobacterium tuberculosis Infection: a key role for IL-1. Cell Rep..

[90] Yao Y (2018). Induction of autonomous memory alveolar macrophages requires T cell help and is critical to trained immunity. Cell.

[91] Netea MG (2020). Defining trained immunity and its role in health and disease. Nat. Rev. Immunol..

[92] Chen J (2023). BCG-induced trained immunity: history, mechanisms, and potential applications. J. Transl. Med..

[93] Verrall AJ (2020). Early clearance of Mycobacterium tuberculosis is associated with increased innate immune responses. J. Infect. Dis..

[94] Joosten SA (2018). Mycobacterial growth inhibition is associated with trained innate immunity. J. Clin. Invest..

[95] Arts RJW (2018). BCG vaccination protects against experimental viral infection in humans through the induction of cytokines associated with trained immunity. Cell Host Microbe.

[96] Eklund D (2014). Human gene variants linked to enhanced NLRP3 activity limit intramacrophage growth of Mycobacterium tuberculosis. J. Infect. Dis..

[97] Mishra BB (2010). Mycobacterium tuberculosis protein ESAT-6 is a potent activator of the NLRP3/ASC inflammasome. Cell Microbiol..

[98] Welin A, Eklund D, Stendahl O, Lerm M (2011). Human macrophages infected with a high burden of ESAT-6-expressing M. tuberculosis undergo caspase-1- and cathepsin B-independent necrosis. PLoS One.

[99] Kaufmann E (2018). BCG educates hematopoietic stem cells to generate protective innate immunity against tuberculosis. Cell.

[100] Khan N (2020). Tuberculosis reprograms hematopoietic stem cells to limit Myelopoiesis and impair trained immunity. Cell.

[101] Williams, A. & Orme I. M. Animal models of tuberculosis: an overview. *Microbiol. Spectr*. **4**, 10.1128/microbiolspec.TBTB2-0004-2015. (2016). 10.1128/microbiolspec.TBTB2-0004-2015 27726810

[102] Orme IM (2003). The mouse as a useful model of tuberculosis. Tuberculosis.

[103] Beamer GL, Turner J (2005). Murine models of susceptibility to tuberculosis. Arch. Immunol. Ther. Exp..

[104] Medina E, North RJ (1998). Resistance ranking of some common inbred mouse strains to Mycobacterium tuberculosis and relationship to major histocompatibility complex haplotype and Nramp1 genotype. Immunology.

[105] Pichugin AV, Yan BS, Sloutsky A, Kobzik L, Kramnik I (2009). Dominant role of the sst1 locus in pathogenesis of necrotizing lung granulomas during chronic tuberculosis infection and reactivation in genetically resistant hosts. Am. J. Pathol..

[106] Kramnik I, Beamer G (2016). Mouse models of human TB pathology: roles in the analysis of necrosis and the development of host-directed therapies. Semin. Immunopathol..

[107] Driver ER (2012). Evaluation of a mouse model of necrotic granuloma formation using C3HeB/FeJ mice for testing of drugs against Mycobacterium tuberculosis. Antimicrob. Agents Chemother..

[108] Churchill GA (2004). Complex trait C. The Collaborative Cross, a community resource for the genetic analysis of complex traits. Nat. Genet..

[109] Smith, C. M. et al. Host-pathogen genetic interactions underlie tuberculosis susceptibility in genetically diverse mice. *Elife*. **11**, Epub 2022/02/04. 10.7554/eLife.74419. (2022). 10.7554/eLife.74419 PMC8846590 35112666

[110] Smith, C. M. et al. Functionally overlapping variants control tuberculosis susceptibility in collaborative cross mice. mBio. 10, Epub 2019/11/28. 10.1128/mBio.02791-19. (2019). 10.1128/mBio.02791-19 PMC6879725 31772048

[111] Smith, C. M., et al. Tuberculosis susceptibility and vaccine protection are independently controlled by host genotype. MBio. **7**, 10.1128/mBio.01516-16. (2016). 10.1128/mBio.01516-16 PMC5030360 27651361

[112] Lai, R., et al. Host genetic background is a barrier to broadly effective vaccine-mediated protection against tuberculosis. *J. Clin. Invest*. 10.1172/JCI167762. (2023). 10.1172/JCI167762 PMC10313364 37200108

[113] Kurtz, S. L., et al. The Diversity outbred mouse population is an improved animal model of vaccination against tuberculosis that reflects heterogeneity of protection. *mSphere*. **5**, 10.1128/mSphere.00097-20. (2020). 10.1128/mSphere.00097-20 PMC7160682 32295871

[114] Niazi MK (2015). Lung necrosis and neutrophils reflect common pathways of susceptibility to Mycobacterium tuberculosis in genetically diverse, immune-competent mice. Dis. Model Mech..

[115] Balasubramanian V, Wiegeshaus EH, Taylor BT, Smith DW (1994). Pathogenesis of tuberculosis: pathway to apical localization. Tube. Lung Dis..

[116] Donald PR (2018). Droplets, dust, and guinea pigs: an historical review of tuberculosis transmission research, 1878–1940. Int J. Tuberc. Lung Dis..

[117] Jacobs AL (1941). Infective dose in pulmonary tuberculosis. Tubercle.

[118] Plumlee CR (2021). Ultra-low dose aerosol infection of mice with Mycobacterium tuberculosis more closely models human tuberculosis. Cell Host Microbe.

[119] Plumlee C. R. et al. Assessing vaccine-mediated protection in an ultra-low dose Mycobacterium tuberculosis murine model. bioRxiv. Epub 2023/03/31. 10.1101/2023.03.22.533820. (2023). 10.1371/journal.ppat.1011825 PMC10703413 38011264

[120] Daniel TM (2006). The history of tuberculosis. Respir. Med.

[121] Bustamante J, Boisson-Dupuis S, Abel L, Casanova JL (2014). Mendelian susceptibility to mycobacterial disease: genetic, immunological, and clinical features of inborn errors of IFN-gamma immunity. Semin. Immunol..

[122] Abel L (2018). Genetics of human susceptibility to active and latent tuberculosis: present knowledge and future perspectives. Lancet Infect. Dis..

[123] Bell LCK, Noursadeghi M (2018). Pathogenesis of HIV-1 and Mycobacterium tuberculosis co-infection. Nat. Rev. Microbiol..

[124] Peters J. M. et al. Protective intravenous BCG vaccination induces enhanced immune signaling in the airways. bioRxiv. 2023:2023.07.16.549208. 10.1101/2023.07.16.549208.

[125] Kamali AN (2019). A role for Th1-like Th17 cells in the pathogenesis of inflammatory and autoimmune disorders. Mol. Immunol..

[126] Li W (2023). A single-cell view on host immune transcriptional response to in vivo BCG-induced trained immunity. Cell Rep..

[127] Lee A., et al. Integrated organ immunity: antigen-specific CD4-T cell-derived IFN-γ induced by BCG imprints prolonged lung innate resistance against respiratory viruses. bioRxiv. 2023:2023.07.31.551354. 10.1101/2023.07.31.551354.

[128] NIAID. Immune Mechanisms of Protection Against Mycobacterium tuberculosis Centers (IMPAc-TB) 2019. 10.3389/fimmu.2024.1429250 PMC11493620 39439805

[129] Brubaker D. K., et al. An interspecies translation model implicates integrin signaling in infliximab-resistant inflammatory bowel disease. *Sci Signal*. **13**, 10.1126/scisignal.aay3258. (2020). 10.1126/scisignal.aay3258 PMC7459361 32753478

[130] Suarez-Lopez L. et al. Cross-species transcriptomic signatures predict response to MK2 inhibition in mouse models of chronic inflammation. *iScience*. **24**, 10.1016/j.isci.2021.103406. (2021). 10.1016/j.isci.2021.103406 PMC8609096 34849469

[131] Mott D (2023). High Bacillary Burden and the ESX-1 Type VII Secretion System Promote MHC Class I Presentation by Mycobacterium tuberculosis-Infected Macrophages to CD8 T Cells. J. Immunol..

[132] Yang JD (2018). Mycobacterium tuberculosis-specific CD4+ and CD8+ T cells differ in their capacity to recognize infected macrophages. PLoS Pathog..

[133] Sutiwisesak R (2020). A natural polymorphism of Mycobacterium tuberculosis in the esxH gene disrupts immunodomination by the TB10.4-specific CD8 T cell response. PLoS Pathog..

[134] Woodworth JS (2014). Protective CD4 T cells targeting cryptic epitopes of Mycobacterium tuberculosis resist infection-driven terminal differentiation. J. Immunol..

